# Continuous flow hydrogenation using polysilane-supported palladium/alumina hybrid catalysts

**DOI:** 10.3762/bjoc.7.83

**Published:** 2011-05-31

**Authors:** Hidekazu Oyamada, Takeshi Naito, Shū Kobayashi

**Affiliations:** 1 Department of Chemistry, School of Science and Graduate School of Pharmaceutical Sciences, The University of Tokyo, Hongo, Bunkyo-ku, Tokyo 113-0033, Japan

**Keywords:** flow chemistry, hydrogenation, polysilane, palladium, reduction

## Abstract

Continuous flow systems for hydrogenation using polysilane-supported palladium/alumina (Pd/(PSi–Al_2_O_3_)) hybrid catalysts were developed. Our original Pd/(PSi–Al_2_O_3_) catalysts were used successfully in these systems and the hydrogenation of unsaturated C–C bonds and a nitro group, deprotection of a carbobenzyloxy (Cbz) group, and a dehalogenation reaction proceeded smoothly. The catalyst retained high activity for at least 8 h under neat conditions.

## Findings

Catalytic hydrogenation is one of the most important methods for the reduction of C–C double and triple bonds, and other functional groups. Heterogeneous catalysts including Pd/C, Pt/C and Pd/SiO_2_ are commonly used in reduction reactions both in research and industrial environments because of the ease of separation of the catalysts and products. However, the contamination of products as a result of leaching of metals from supports as well as the decreased catalytic activity, are both serious problems with the use of conventional heterogeneous catalysts. To address these problems, we recently developed novel methods for the immobilization of metal and non-metal catalysts on supports. This involved the development of microencapsulated (MC) and polymer-incarcerated (PI) catalysts, which have high catalytic activity without causing metal leaching [1].

Heterogeneous catalytic hydrogenation in a batch system has recently been applied to continuous flow hydrogenation systems for high-throughput synthesis [2–10]. There are several problems associated with conducting heterogeneous catalytic hydrogenation in a batch system. These include the necessity for filtration of hydrogen-saturated pyrophoric catalysts from flammable solvents, the possible requirement to use hydrogen gas under high pressure, difficulties in the re-use of catalysts (or inability for their re-use), the need for vigorous stirring to achieve catalytic activity, and the possible necessity for longer reaction times, which may lead to undesirable side reactions or harsh reaction conditions. Continuous flow systems have the potential to overcome these problems and disadvantages. In our laboratory, PI catalysts were applied to tri-phase hydrogenation reactions in a microreactor system and led to markedly shorter reaction times [11]. While the productivity of a single microreactor is low, increasing the number of reactors (“numbering up”) could readily and substantially increase production.

We also recently reported novel transition metal catalysis involving the immobilization of Pd or Pt onto polysilane supports by the microencapsulation method [12]. The Pd catalyst (Pd/PSi) had high activity with no or very little leaching of Pd, and it could be recovered and re-used in hydrogenation reactions in batch systems. We then developed polysilane-supported Pd/metal oxide hybrid catalysts [13] using the PI method (microencapsulation and cross-linking), which were then applied in these microreactor systems [14]. The hybrid catalysts were insoluble, did not swell in any solvent, and were predicted to be applicable to continuous flow reactors. In this study, we investigated hydrogenation reactions of C–C double and triple bonds as well as various other functional groups using continuous flow systems with Pd/(PSi–Al_2_O_3_) catalysts.

**Figure 1:**
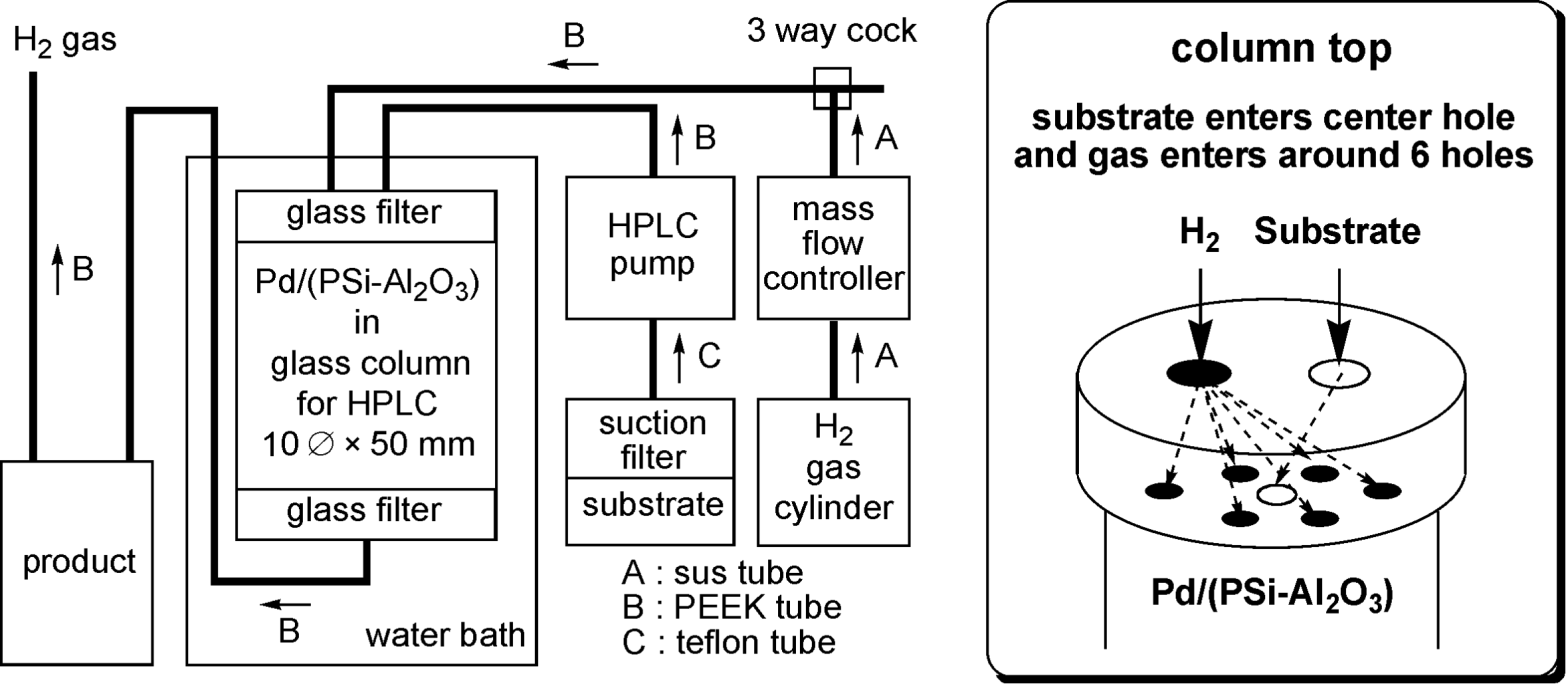
Schematic diagram of the continuous flow reactor (left) and the column top (right).

A schematic diagram of the continuous flow reactor and an image of the top of the column are shown in Figure 1. A high performance liquid chromatography (HPLC) pump was used to feed the substrate into the central hole in the top of the column, which was filled with the Pd/(PSi–Al_2_O_3_) catalyst. Hydrogen gas was introduced into the six holes surrounding the central hole using a mass flow controller. The column was heated in a water bath as required. The substrate reacted with the H_2_ gas inside the column, and the product was collected downstream of the column.

**Scheme 1:**
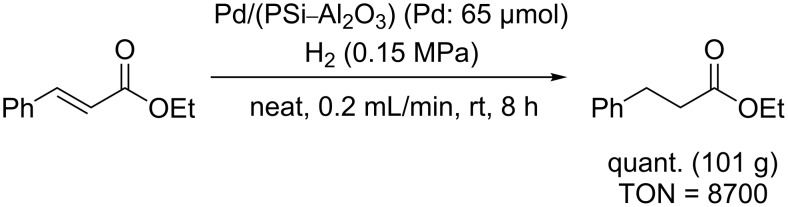
Hydrogenation of ethyl cinnamate.

We initially examined the hydrogenation reaction of ethyl cinnamate (Scheme 1) and collected the product for 8 h without contamination of the starting material. This demonstrated that the catalyst retained high activity for at least 8 h, and the turnover number (TON) reached 8700.

We then investigated the hydrogenation of other substrates. The hydrogenation reactions of C–C double and triple bonds in various substrates are shown in Table 1; these proceeded quantitatively at room temperature under neat conditions. The products were obtained at approximately 10 g/h through the 4 cm^3^ column.

**Table 1:** Hydrogenation of C–C double and triple bonds.

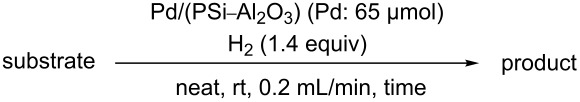				

Entry	Substrate	Time (h)	Product	Yield (TON)

1	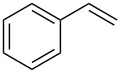	1.5	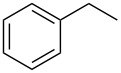	14.9 g (2200)
2	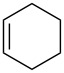	1.5	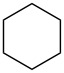	13.9 g (2400)
3	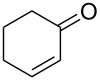	1.5	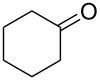	18.0 g (2700)
4	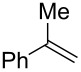	1.5	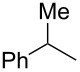	16.3 g (2100)
5	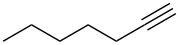	1.7	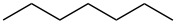	14.9 g (2300)
6	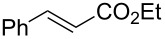	1.5	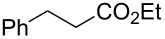	19.4 g (1700)

**Scheme 2:**
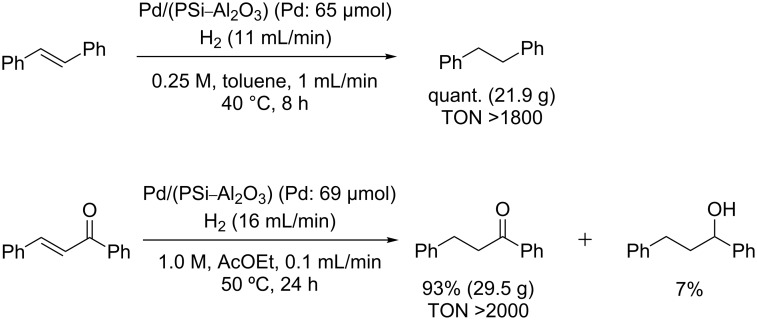
Hydrogenation of *trans*-stilbene and *trans*-chalcone.

The hydrogenation reactions of *trans*-stilbene and *trans*-chalcone, as representative solid substrates (Scheme 2), were also examined. The substrates were dissolved in toluene or ethyl acetate. The reduction of *trans*-stilbene proceeded quantitatively, but an overreaction product was obtained (7% yield) in the reduction of *trans*-chalcone.

**Scheme 3:**
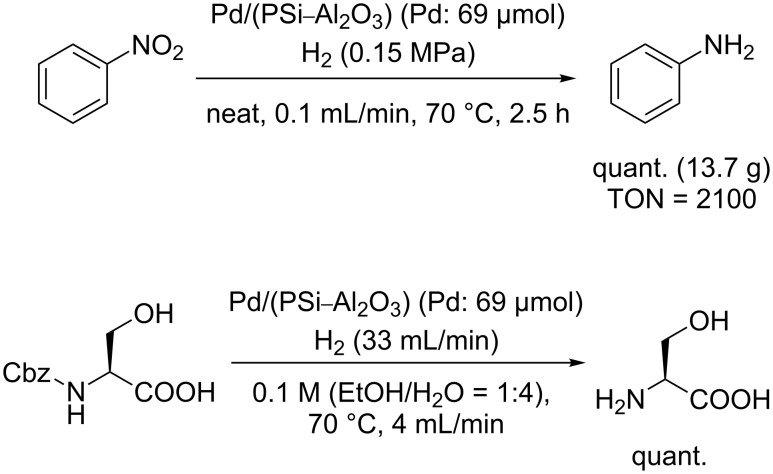
Hydrogenation of nitrobenzene and deprotection of the Cbz group.

We then investigated the hydrogenation of a nitro group (nitrobenzene) and the deprotection of a carbobenzyloxy (Cbz) group (Scheme 3). The hydrogenation reaction of nitrobenzene proceeded quantitatively under neat conditions, and the deprotection of Cbz–Ser also proceeded quantitatively in a mixed solvent system (EtOH/H_2_O 1:4).

**Scheme 4:**
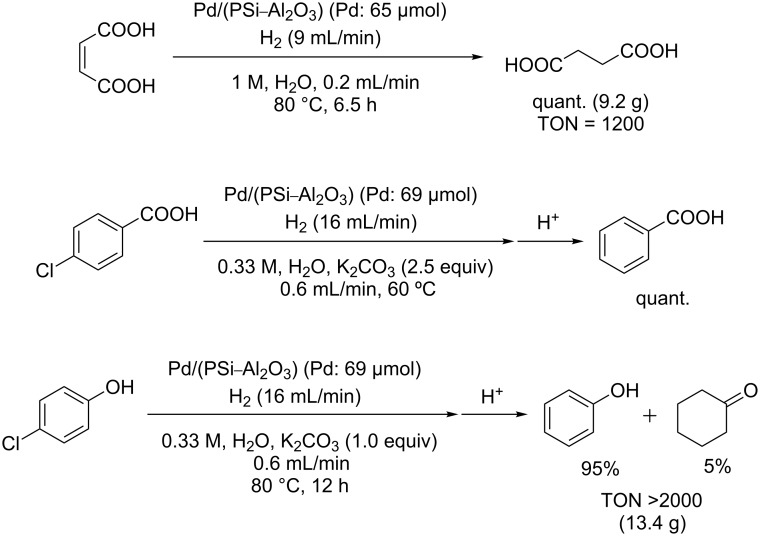
Hydrogenation in water.

Hydrogenation reactions could also be carried out successfully in water (Scheme 4). The reduction of aqueous maleic acid proceeded quantitatively. Dehalogenation of *p*-chlorobenzoic acid in basic aqueous solution also proceeded smoothly and benzoic acid was obtained after acid treatment. In the case of *p*-chlorophenol, the conversion was complete; however, in this case some by-product (5%) was also obtained.

In summary, we developed continuous flow systems for hydrogenation using a Pd/(PSi–Al_2_O_3_) catalyst. Our original Pd/(PSi–Al_2_O_3_) catalysts were successfully used in these systems. In the hydrogenation reactions studied, i.e., the reduction of unsaturated C–C bonds and a nitro group, deprotection of a Cbz group, and a dehalogenation reaction, all proceeded smoothly. The catalysts could be used for a long time, with high activity being retained for at least 8 h under neat conditions. It is noted that in all cases no Pd leaching was detected (ICP). Further studies of the application of these systems to large-scale production are now in progress.

## Experimental

The continuous flow reactor system comprised the following devices (Figure 1): HPLC pump: Shimadzu LC-6AD or Eyela 301. Mass flow controller: Lyntec MC-3000E and RP-300. Column: 10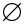 × 50 mm Eyela glass column filled with 4 g of Pd/(PSi–Al_2_O_3_) and equipped on the top with a glass filter (pore size 10 μm), a 7-hole plate (polychlorotrifluoroethylene; PCTFE) and a screw cap, and on the bottom with a glass filter (pore size 10 μm), a 1-hole plate (PCTFE) and a screw cap. Line: A sus tube (outside diameter: 1/16 inch) was used for line A, a PEEK tube (outside diameter: 1/16 inch) was used for line B and a Teflon tube (outside diameter: 3 mm) was used for line C.

Typical procedure for hydrogenation reactions (Table 1, entry 1): Styrene was fed into the column using the HPLC pump, and H_2_ gas was introduced into the column using the mass flow controller. The system was left to stabilize for 30 min, and the product was then sampled over 1.5 h. The sample was analyzed by ^1^H NMR, and the complete conversion of styrene to ethyl benzene was confirmed. ^1^H NMR (500 MHz, CDCl_3_) δ 1.24 (t, *J* = 7.7 Hz, 3H), 2.65 (q, *J* = 7.7 Hz, 2H), 7.15–7.23 (m, 3H), 7.26–7.30 (m, 2H).

Hydrogenation reaction of *trans*-stilbene (Scheme 2): *trans*-Stilbene (Aldrich) in toluene (0.25 M) was fed into the column (maintained at 40 °C in a water bath) using the HPLC pump (1.0 mL/min), and H_2_ gas was introduced into the column using the mass flow controller (11 mL/min). The system was left to stabilize for 30 min. The product was then sampled over 8 h and dried in vacuo. The sample was analyzed by ^1^H NMR, and the full conversion of *trans*-stilbene to 1,2-diphenylethane was confirmed. ^1^H NMR (500 MHz, CDCl_3_) δ 2.92 (s, 4H), 7.17–7.21 (m, 6H), 7.26–7.30 (m, 4H).

Hydrogenation reaction of nitrobenzene (Scheme 3): Nitrobenzene was fed into the column (maintained at 70 °C in a water bath) using the HPLC pump (0.1 mL/min), and H_2_ gas was introduced into the column at a pressure of 0.15 MPa. The system was left to stabilize for 30 min and the product then sampled for 2.5 h. The sample (18.9 g as a mixture of aniline and H_2_O; calculated quantity of aniline = 13.7 g) was analyzed by GC, and the complete conversion of nitrobenzene to aniline was confirmed.

Hydrogenation reaction of *p*-chlorobenzoic acid (Scheme 4): *p*-Chlorobenzoic acid in H_2_O (0.33 M, including 2.5 equiv of K_2_CO_3_) was fed into the column (maintained at 60 °C in a water bath) using the HPLC pump (0.6 mL/min) and H_2_ gas was introduced into the column using the mass flow controller (16 mL/min). The system was left to stabilize for 30 min and the product was then sampled for 8 min. Aqueous HCl (1 N) was added to the mixture, which was then extracted twice with AcOEt. The combined organic layers were dried in vacuo. The sample was analyzed by ^1^H NMR, and complete dehalogenation of the Cl group was confirmed. ^1^H NMR (500 MHz, CDCl_3_) δ 7.46–7.50 (m, 2H), 7.60–7.63 (m, 1H), 8.12–8.14 (m, 2H).
